# Orchestration of H3K27 methylation: mechanisms and therapeutic implication

**DOI:** 10.1007/s00018-017-2596-8

**Published:** 2017-07-17

**Authors:** Mei-Ren Pan, Ming-Chuan Hsu, Li-Tzong Chen, Wen-Chun Hung

**Affiliations:** 1 Graduate Institute of Clinical Medicine, College of Medicine, Kaohsiung Medical University, Kaohsiung, 807 Taiwan; 2 National Institute of Cancer Research, National Health Research Institutes, Tainan, 704 Taiwan; 3 Division of Hematology/Oncology, Department of Internal Medicine, National Cheng Kung University Hospital, Tainan, 704 Taiwan; 4 Graduate Institute of Medicine, College of Medicine, Kaohsiung Medical University, Kaohsiung, 804 Taiwan

**Keywords:** Histone modification, Gene mutation, Polycomb repression complex 2, Epigenetic drugs

## Abstract

Histone proteins constitute the core component of the nucleosome, the basic unit of chromatin. Chemical modifications of histone proteins affect their interaction with genomic DNA, the accessibility of recognized proteins, and the recruitment of enzymatic complexes to activate or diminish specific transcriptional programs to modulate cellular response to extracellular stimuli or insults. Methylation of histone proteins was demonstrated 50 years ago; however, the biological significance of each methylated residue and the integration between these histone markers are still under intensive investigation. Methylation of histone H3 on lysine 27 (H3K27) is frequently found in the heterochromatin and conceives a repressive marker that is linked with gene silencing. The identification of enzymes that add or erase the methyl group of H3K27 provides novel insights as to how this histone marker is dynamically controlled under different circumstances. Here we summarize the methyltransferases and demethylases involved in the methylation of H3K27 and show the new evidence by which the H3K27 methylation can be established via an alternative mechanism. Finally, the progress of drug development targeting H3K27 methylation-modifying enzymes and their potential application in cancer therapy are discussed.

## Introduction

Genomic DNA and the associated histone proteins form the nucleosomes, the building blocks of eukaryotic chromatin. The dynamic change of chromatin structure and gene transcription is mainly controlled by epigenetic regulation, including DNA methylation and histone modification. Methylation of DNA occurs at the cytosine residues of the CpG dinucleotides in vertebrates, and the concept that this modification serves as an important epigenetic marker was first proposed by two elegant studies [1, 2]. Generation of the 5′-methylcytosine (5′-mc) by DNA methyltransferases (DNMTs) creates a specific marker, which could be recognized by methyl-CpG binding domain proteins (MBDs) and methyl-CpG binding zinc-finger proteins of the Kaiso family. These proteins recruit histone deacetylases (HDACs) and remove the acetyl group from histone proteins, which lead to downregulation of gene transcription [3–5]. 5′-mc was considered an extremely stable marker for a long time until identification of the ten-eleven translocation (*TET*) genes was made [6]. This gene family consists of three members (*TET1* to *3*), and the encoded proteins exhibit dioxygenase activity that may catalyze the removal of the methyl group from 5′-mc in an Fe(II)- and α-ketoglutarate-dependent manner [7–9]. TET proteins convert 5′-mc to 5′-hydroxymethylcytosine (5′-hmc) first and further catalyze 5′-hmc to 5′-formylcytosine (5′-fc) and 5′-carboxylcytosine (5′-cc). Finally, 5′-cc is recognized and cleaved by thymidine DNA glycosylase and the base-excision repair system. Compared to DNA methylation, post-translational modification of histone proteins is more complicated due to the involvement of (1) the histone proteins, (2) the types of modification, (3) the position of modification, (4) the degree of modification and (5) the crosstalk between different modifications. A brief review of the “histone code” hypothesis and the progress of our understanding in the functional significance of histone modification are summarized below.

### The language of histone modification

The hypothesis of “histone code” was proposed by Strahl and Allis to describe the concept that distinct chemical modifications on one or more histone tails may form readable codes that could be recognized by specific proteins to elicit distinct downstream events like transcriptional activation, gene silencing, DNA repair, etc., to determine the biological consequences during cell fate decision, tissue organization and development [10, 11]. As aforementioned, the impact of histone modifications is based on several factors. First, for the histone proteins, four types exist in the nucleosomal core being histone 2A (H2A), H2B, H3 and H4. Among them, H3 receives the most extensive modifications and the biological significance of some modifications have been well characterized [12, 13]. For instance, acetylation of lysine 14 of H3 (H3K14), methylation of lysine 4 (H3K4) and phosphorylation of serine 10 (H3S10) all imply the activation of gene transcription [14–16]. Conversely, methylation of H3K9 and H3K27 is frequently associated with gene repression [17, 18]. Thanks to the development of mass spectrometry-based techniques, more histone modifications have been identified and the high-throughput analysis of combinational histone codes have been established [19]. In addition to the core histone molecules, modification of other histone proteins like H1, H3.3 or their variants and the readout of these modifications are still under intensive investigation. Recent studies show chromosome mis-segregation may trigger the phosphorylation of serine 31 on H3.3 and increase the accumulation of nuclear p53 in the aneuploid daughters to prevent cell cycle progression [20]. Second, for the types of modification, different modifications on the same residue of histone proteins generally yield different outcomes. As an example, acetylation of H3K9 is a gene activation marker, while methylation of this residue is a typical repression marker [21, 22]. Third, for the position of modification, the same modification occurring at different residues of a single histone protein may also give different outcomes. For instance, methylation on H3K4 usually increases gene expression while methylation on H3K9 always decreases gene expression. Fourth, for the degree of modification, a number of histone modifications like phosphorylation only add a single chemical group into a specific residue of histone proteins; however, several chemical modifications like methylation could introduce multiple chemical groups on the same residue simultaneously. For example, lysine and arginine of histone proteins could undergo mono-methylation (me1), di-methylation (me2) and tri-methylation (me3) [23, 24]. Interestingly, the degree of methylation also has distinct impact on gene expression. Me2 and me3 of H3K9 are found in silenced genomic regions [25]. On the contrary, me1 of H3K9 is frequently detected in the promoter region of actively transcribed genes [26, 27]. Fifth, for the crosstalk between different modifications, Daujat et al. [28] demonstrated that estrogen stimulation induced a consequent acetylation and arginine methylation of H3 to activate the transcription of downstream target genes suggesting the combinatory histone modifications modulate gene expression. Another example is the interplay between H3S10 phosphorylation and H4K14 acetylation established a histone code that enhances transcription elongation [16]. In this review, we will focus on how cells orchestrate specific histone methylation markers by multiple enzymatic complexes and different mechanisms.

### Histone methylation

The addition of the methyl group to lysine and arginine residues of histone proteins is carried out by histone lysine methyltransferases and histone arginine methyltransferases, respectively [29, 30]. Because these enzymes methylate both histone and non-histone substrates, they are also named protein lysine methyltransferases (KMTs) and protein arginine methyltransferases (PRMTs). In this review, we focus on the KMTs and their roles in the regulation of chromatin structure and gene transcription. KMTs use *S*-adenosyl-*l*-methionine (SAM) as the methyl donor and transfer one to three methyl groups to the recipient residue of protein substrates [31]. The catalytic reaction results in mono-, di- and tri-methylated lysine and elicits distinct outputs depending on the position of the residue and the degree of methylation.

Up to date, two families of KMTs divided on the basis of catalytic domain have been reported. Most of the KMTs belong to the suppressor of variegation 3-9 (Su(var)3-9), Enhancer of zeste (E(z)) and Trithorax (SET) family that contains a unique functional SET domain originally found in *Drosophia* polycomb proteins [32–34]. The SET domain is a 130-140 amino acid sequence which composes the SAM and substrate binding sites, and an intra-molecular interacting salt bridge that may determine the product specificity of these methyltransferases [35–37]. From a functional view, the SET KMTs can be divided as repressive or activating KMTs, depending on the residue that is methylated and the degree of methylation. The KMTs that introduce the methylation on H3K9, H3K27 and H4K20 are the main repressive KMTs while the KMTs that target H3K4, H3K14 and H3K36 are considered as activating KMTs [37]. However, as aforementioned, the degree of methylation is another key factor that determines the outcome of a methylated histone residue on gene transcription. The addition of one methyl group to H3K27 frequently increases the expression of target genes while the inclusion of three methyl groups at the same site always silences gene transcription [38]. From a structure point of view, the KMTs can be divided into several subfamilies according to the sequence homology. These subfamilies are (1) the Su(var)3-9 (SUV39) family that includes six members KMT1A to 1F, (2) the Enhancer of Zeste Homolog (EZH) family that contains EZH1 and EZH2, (3) the SET1 family that includes MML1 (mixed-lineage leukemia 1) to MLL4 and SET1 and SET1L, (4) the SET2 family that contains NSD1 (nuclear receptor binding SET domain protein 1) to NSD3 and KMT2H, (5) the RDI-BF1 and RIZ homology domain containing (PRDM) family, (6) the SMYD (SET and MYND domain) family and (7) the other SET family that contains KMT5A to 5C and SET7/9 [39, 40].

Among them, the PRDM family is unique and is defined on a specific protein domain highly conserved at the N-terminal region that is co-shared by the positive regulatory domain I-binding factor 1 (PRDI-BF1) and retinoblastoma protein-interacting zinc finger gene 1 (RIZ1). The protein domain was named as the PR (PRDI-BF1-RIZ1 homologous) domain and was found to be structurally related to the SET domain. Currently, at least 16 PRDM genes have been identified [41]. However, the KMT activity is only clearly demonstrated in several members such as PRDM2, PRDM8, PRDM9 and PRDM16 [42, 43]. The biological function of PRDMs on histone methylation and gene transcription awaits further characterization. Another family of KMTs is the disrupter of telomeric silencing 1-like (DOT1L). The chromosome end of the yeast *Saccharomyces cerevisiae* exhibits a recessive chromatin structure known as telomeric silencing [44]. By using a genetic screening approach, Singer et al. studied genes whose overexpression might affect repression status of chromatin, and identified *Dot1* as an effector gene [45]. However, the function of Dot1 was not known at that time. Human *DOT1L* gene was identified in 2002 as the mammalian homologue of yeast *Dot1* that exhibits histone methyltransferase activity without the SET domain [46, 47]. Currently, DOT1L is the only enzyme found in mammalian cells to catalyze me1, me2 and me3 of H3K79.

The incorporated methyl group on histone proteins can be removed by demethylases. Similar to KMTs, two classes of lysine demethylases (KDMs) with distinct catalytic mechanisms have been described in cells [48–50]. The first class includes lysine-specific demethylase (LSD1, also known as KDM1A) and LSD2 (KDM1B). These two enzymes are amine oxidases and catalyze the demethylation reaction via generation of an imine intermediate [51, 52]. The second class is a large group of histone demethylases with a unique Jumonji-C (JMJC) domain. Up to date, more than 30 members of JMJC demethylases have been reported and these members can be divided into seven sub-families based on their domain homology [53–56]. Unlike LSD1 and 2, JMJC demethylases exhibit dioxygenase activity and remove the methyl groups from lysine in an iron and α-ketoglutarate-dependent fashion [57, 58].

### Regulation of H3K27 methylation

One of the complexities of histone methylation is that the methylation status of each lysine residue is orchestrated by multiple protein complexes. Generally, we use the terms “writer” and “eraser” to describe the enzymes that add and remove the methyl groups on histone proteins, respectively. Here, we introduce the mechanism as to how cells control the methylation of H3K27.

### Writers-from mono-methylation (me1) to tri-methylation (me3)

The enzyme-mediated me1 of H3K27 has been a matter of debate for decades, and recent data suggest differences between various species. In *Arabidopsis*, two novel H3K27me1 methyltransferases ATXR5 and ATXR6 were reported by Jacob et al. [59]. ATXR5 and ATXR6 proteins contain divergent SET domains and functional inactivation of these two genes lead to a significant reduction of H3K27me1. However, it should be noted that double mutant of *atxr5* and *atxr6* did not completely abolish H3K27me1, suggesting the involvement of other methyltransferases in the establishment of this epigenetic marker. In the unicellular eukaryote Tetrahymena thermophila, TXR1, a SET domain protein and a homolog of ATXR5 and ATXR6, is the most important methyltransferase for H3K27me1 because deletion of *TXR1* in Tetrahymena thermophila reduced H3K27me1 by at least 80% [60]. Again, the incomplete inhibition of H3K27me1 suggested the existence of other me1 methyltransferase or a compensatory pathway to modulate the me1 of H3K27 when *TXR1* is depleted.

The first enzyme reported in mammalian cells to introduce me1 on H3K27 was EZH1, a homolog of the Drosophila EZ protein [61]. Two ZH homologs EZH1 and EZH2 are identified in mammalian cells and these two molecules may exist in different polycomb repression complex 2 (PRC2) complexes. The PRC2 complex comprises four core subunits including EZH2 (or EZH1), Suppressor of Zeste 12 (SUZ12), Embryonic Ectoderm Development (EED) and Retinoblastoma protein associated protein 46/48 (RbAp46/48) and this complex has been shown to be involved in the methylation of H3K27 [62–64]. In addition to the core components, several associated molecules including AE binding protein 2 (AEBP2), Jumonji and AT-rich interaction domain containing 2 (JARID2), PHD finger protein 19 (PHF19), polycomb-like proteins (PCLs) and the long intergenic noncoding RNA *HOTAIR* may play crucial roles in the regulation of complex recruitment and enzymatic activity [65–69]. In *Ezh2* ^−/−^ embryonic stem cells (ESCs), H3K27me2 and H3K27me3 were dramatically reduced [61]. However, only a partial decrease of H3K27me1 was found. Knockdown of *Ezh1* in *Ezh2* ^−/−^ ESCs totally abolished H3K27me1, suggesting that EZH1 could be the major enzyme responsive for the introduction of this epigenetic mark. A subsequent study also demonstrated that the PRC2 activity was required for the genome-wide deposition of H3K27me1 in ESCs although it was not specifically clarified whether this was mediated by EZH1 or EZH2 [70]. Other potential H3K27me1 methyltransferases are two closely related enzymes G9a (also known as euchromatic histone lysine methyltransferase 2, EHMT2) and Glp (EHMT1). Recent studies have clearly shown that G9a and Glp are the major H3K9 methyltransferases in vitro and in vivo [71–73]. Although G9a had been shown to methylate H3K9 and H3K27 in vitro [74], its role in the in vivo methylation of H3K27 was not demonstrated until 2011 [75]. However, Mozzetta et al. [72] showed that the expression level of G9a or Glp was not changed in *Ezh2* ^−/−^ or *Eed* ^−/−^ mouse ESCs and the depletion of G9a and Glp activity also did not affect the methylation status of H3K27 in these cells, raising the question whether G9a and Glp only directly methylated H3K27 under specific circumstances.

The main writers for H3K27me2 and H3K27me3 in plants and animals are the PRC2 complexes. Three EZ homologs identified in *Arabidopsis* are MEDEA (MEA), CURLY LEAF (CLF) and SWINGER (SWN) [76]. Level of H3K27me2 and H3K27me3 was reduced in *clf swn* mutants [77]. In addition, these proteins have been reported to suppress gene expression by increasing H3K27me3 [78, 79]. In Tetrahymena thermophila, three genes *EZL1*, *EZL2* and *EZL3* are found to be the EZ homologs [80, 81]. Among them, *EZL2* is expressed at higher level and is required for the introduction of me2 and me3 of H3K27 [82]. Two pioneer studies established the role of PRC2 in the high-level methylation of H3K27 in mammalian cells. Pasini et al. [62] demonstrated a specific loss of H3K27me2 and H3K27me3 in the *Suz12*-null embryos, suggesting the regulation of H3K27 methylation by SUZ12-containing PRC2 complexes. Montgomery et al. [63] reported that knockout of another PRC2 component EED also induced a global reduction of H3K27me2 and H3K27me3. However, they also point out a decrease of H3K27me1 in *Eed*-deficient embryos. Up to date, PRC2 seems to be the only, or the most important, methyltransferase for H3K27 in mammalian cells.

Although the association of H3K27me3 and gene repression has been extensively studied, the functional role of H3K27me1 and H3K27me2 in gene regulation has only recently been revealed. Ferrari et al. [38] demonstrated that PRC2-mediated H3K27me1 is enriched within transcribed genes in ESCs. More importantly, they found that the deposition of H3K27me1 is regulated by H3K36 trimethylation generated by SET domain containing 2 (SETD2) and is associated with gene activation. The co-existence of H3K27me1 and H3K36me3 may lead to the high mobility of histones and nucleosomes with loose chromatin structure for transcriptional initiation and elongation. Conversely, H3K27me3 is mutually exclusive with the gene activation marks H3K36me3 and H3K4me3 in the human genome and creates a compact heterochromatin that prevents the binding of transcriptional machinery [83, 84]. H3K27me2 presenting in a high proportion of H3 protein is detected in large chromatin domains and plays a role in the control of enhancer fidelity by avoiding the unscheduled activation of specific enhancers.

Because deletion of the *EED* gene does not completely abolish the recruitment of PRC2 to chromatin and the methylation of H3K27, the existence of other mediators for PRC2 recruitment is suggested. A previous study demonstrated that the nucleosome remodeling and deacetylase (NuRD) complex facilitates the chromatin binding to repress gene transcription [85]. Recently, an elegant study revealed the underlying mechanism by which the NuRD complex promotes PRC2 recruitment [86]. Wei et al. showed that metastasis-associated member 2 (MTA2), a component of the NuRD complex, binds to the unmodified H3 via the SANT domain and directly interacts with EZH2 via the BAH domain to recruit the PRC2 complex to silence the transcription of several suppressors of the mechanistic target of rapamycin (mTOR) pathway. This results in the inhibition of autophagy and the activation of the mTOR signaling and provides a molecular basis as to how epigenetic regulation controls autophagy induction and tumorigenesis.

### Erasers-from tri-methylation to mono-methylation

For a long time, H3K27me3 was considered a stable epigenetic marker that could not be removed. This hypothesis was changed by the identification of the JMJC-domain proteins, the ubiquitously transcribed tetratricopeptide repeat X chromosome (UTX, now named as KDM6A) and Jumonji D3 (JMJD3, also known as KDM6B) as H3K27me3 demethylases in 2007 [53–56]. H3K27me3 level was high in ESCs and was rapidly decreased during embryogenesis and stem cell differentiation. By studying ESC differentiation, Agger et al. [57] showed that UTX and JMJD3 are key enzymes for the demethylation of H3K27me3. De Santa et al. [54] found that inflammatory cytokines triggered transdifferentiation of macrophages that was associated with the reduction of H3K27me3 and the de-repression of reprogramming genes. They identified JMJD3 as an inducible enzyme to remove the H3K27me3 marker to alter macrophage plasticity in response to the extracellular microenvironment. Lan et al. [55] demonstrated that UTX and JMJD3 regulated the expression of many homeobox (*HOX*) genes and controlled anterior–posterior development of zebrafish. Lee et al. [56] also identified UTX as a H3K27me2 and H3K27me3 demethylase and might associate with mixed-lineage leukemia (MLL) 2/3 complex to activate gene transcription by coupling demethylation of H3K27 and methylation of H3K4. Results of these studies support the notion that KDM6A and 6B are the major erasers of H3K27me3.

Although KDM6A and 6B could demethylate H3K27me1 in vitro, these two enzymes do not show H3K27me1 demethylase activity in vivo. KDM7A (also known as KIAA1718) may be the enzyme responsible for the demethylation of H3K27me1 in cells. Two studies reported in early 2010 showed that KDM7A is a dual-specificity histone demethylase that could induce demethylation of H3K9me2 and H3K27me2 simultaneously [87, 88]. Study of substrate preference suggested that KDM7A could also demethylate H3K27me1 at least in vitro [89].

### Crosstalk between histone marks to modulate H3K27 methylation

The crosstalk between PRC2-mediated H3K27 methylation with other histone marks was only proposed several years ago. Yuan et al. [90] found that H3K27me3 was rarely co-existent with H3K36me2 or H3K36me3 in cells and the preexisting H3K36 significantly suppressed the methylation of H3K27 by the PRC2 complex in vitro and in vivo. More recently, Mozzetta et al. [72] reported another novel crosstalk between H3K27 and H3K9. They found that G9a and PRC2 complex could physically interact with each other and G9a activation could increase the recruitment of PRC2 complex to the promoters of a set of developmental genes. Because the methylation of H3K27 and H3K9 plays critical roles in gene silencing, the cooperation between G9a and PRC2 may constitutively repress specific target genes to regulate cell fate and function.

Two studies by using quantitative proteomics approach revealed other potential histone modifications that may link with H3K27 methylation. Zhang et al. [81] found that the depletion of *TRX1* and *EZL2* led to deficiency of H3K27 methylation and hyper-acetylation in H2A, H2A.Z and H4, suggesting crosstalk between these epigenetic markers. Because H3K27 methylation is generally associated with heterochromatin, the decrease of H3K27 methylation might facilitate chromatin decondensation and increase the accessibility of histone proteins to acetyltransferases. However, hyper-acetylation was only detected in specific residues, pointing out that the crosstalk of H3K27 methylation with these epigenetic marks is not random. Yu et al. [91] investigated the pattern of the combinational K27/K36 epigenetic codes in histone proteins and found a significant difference in the modifications of these two marks in H3 variants. More interestingly, the distribution of H27/K36 modifications was also different in different organs, implying the crosstalk between histone codes might elicit a context-dependent effect during development and terminal differentiation.

**Fig. 1:**
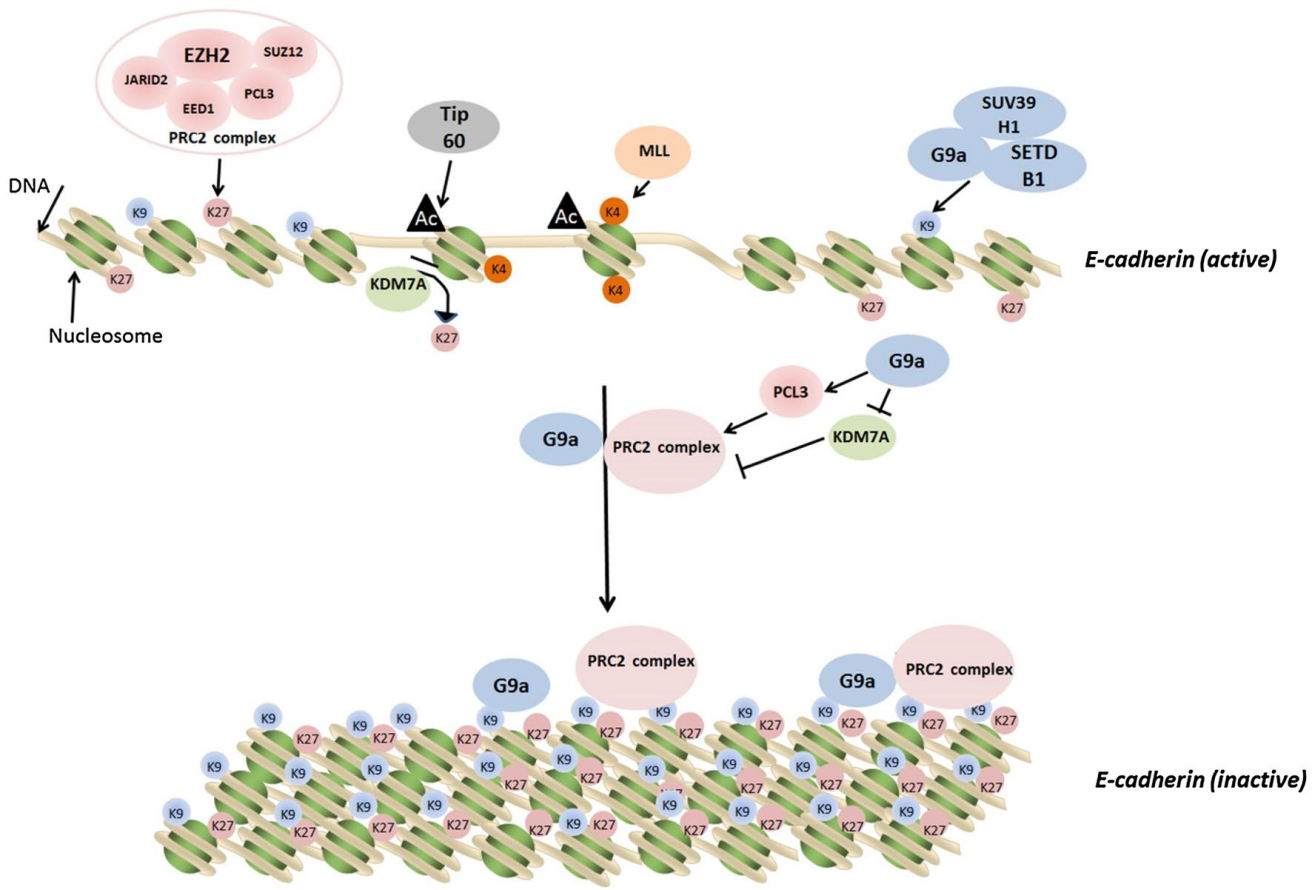
Dual regulation of histone methylation on H3K9 and H3K27 by G9a. Two proposed mechanisms explain the coordination of H3K9 and H3K27 methylation by the histone methyltransferase G9a to silence an important epithelial marker and tumor suppressor gene *E*-*cadherin*. In the first mechanism, G9a physically couples with EZH2 to form a super repression complex to methylate H3K9 and H3K27 simultaneously [72]. In the second mechanism, G9a directly methylates H3K9 and indirectly increases H3K27 methylation via epigenetically upregulating the PCL3 gene to promote the chromatin recruitment of PRC2 and downregulating the KDM7A gene to attenuate the demethylation [67]

As demonstrated by Mozzetta et al. [72], the crosstalk between different histone modifications could be orchestrated by physical interaction and functional cooperation between various histone-modifying enzymatic complexes. The list of such regulatory complexes is increasing. For example, the H3K27 demethylase KDM6A and the H3K4 methyltransferase MLL4 have been shown to coordinately regulate genes associated with growth and invasion of breast cancer cells [92]. By using immunoprecipitation and mass spectrometry analysis, Shi et al. [93] showed that the H3K9 demethylase JMJD2B formed a complex with the H3K4 methyltransferase mixed-lineage leukemia 2 (MLL2), and this complex interacted with estrogen receptor α (ER-α) to activate ER-α-dependent gene transcription. Recently, we found another mechanism by which histone-modifying enzymes modulate epigenetic marks via an indirect manner [67]. We demonstrated that the H3K9 methyltransferase G9a could upregulate the expression of PCL3 to enhance the recruitment of the PRC2 complex to the promoter of *E*-*cadherin*. Importantly, G9a directly repressed the expression of the H3K27 demethylase KDM7A and reduced its binding to the *E*-*cadherin* gene promoter. Collectively, G9a acts via a dual regulation of the methyltransferase (PRC2) and the demethylase (KDM7A) to increase the H3K27 methylation to downregulate *E*-*cadherin* expression (Fig. 1). Our results provide a molecular basis by which a methyltransferase regulates other epigenetic-modifying enzymes to modulate specific histone codes. However, such regulation may only be involved in the control of a set of target genes to fine-tune specific gene expression to adapt cellular response to extracellular stimuli. A genome-wide study will be needed to identify the target genes controlled by this indirect regulatory mechanism during development, differentiation, and tumorigenesis.

### Mutations in H3K27 methylation modifiers in cancers and in precancerous lesions

Since the methylation status of H3K27 has great impact on gene expression and cellular function, it is predictable that mutations in the methyltransferases or demethylases that control this histone marker will globally change H3K27 methylation in cells and may be generally found in cancers or in precancerous lesions. Mounting evidence has indeed shown a number of mutations in these histone modifiers. We summarize the mutations reported in different studies in Table 1.

**Table 1:** Mutations in the H3K27 methylation modifiers

Gene	Tumor type	Mutations	Functional alteration	References
*EZH2*	Hematological malignancies (B-cell lymphoma, myelodysplastic syndrome, T-ALL, follicular lymphoma etc.)	Y641F, A677G, A687V, I646F, R679H, N688Y, R690H, Y733X, etc.	Gain- or loss-of-function	[95–105]
	Parathyroid tumor Melanoma	Y641N, R216Q, A226V, G464E, K515R, Y646F, Y646N, Y646S, G709S etc.	Gain-of-function Gain-of-function or non-synonymous	[106–109]
	Hepatocellular carcinoma Pediatric cancer	G553C, S695L, A682G, N675K, Y646C etc.	Gain-of-function or unknown	[110]
*EED*	Myelodysplastic syndrome myeloproliferative neoplasm	G255D	Loss-of-function	[103]
	T-ALL	S259F, N286sf, R436C	Unknown	[111]
*SUZ12*	Myelodysplastic syndrome myeloproliferative neoplasm	F603L, D605V, E610G	Loss-of-function	[103]
	T-ALL	S369sf, S568fs	Unknown	[111]
*KDM6A*	Bladder cancer	D336G, P996R, Y1114S, Y1173N, G1223D etc.	Loss-of-function Unknown	[112–114]
	Multiple cancers (AML colon cancer, renal cell carcinoma etc.)	R172X, E216X, Q333X, Q541X, Q667X, etc.	Loss-of-function Unknown	[115]
	Adenoid cystic carcinoma	T1002A, G1140E, I1267V, G1335L, L1375P	Loss-of-function Unknown	[116]
	Pancreatic cancer	P428S, D1216N, E1290K, A269fs, L231fs, L1288fs etc.	Loss-of-function or Unknown	[117, 118]
	T-ALL	Q692*	Unknown	
*KDM6B*	T- and NK-cell post-transplant lymphoproliferative disorders	P1682S, L251S	Unknown	[119]

T-ALL, T-cell acute lymphoblastic leukemia; AML, acute myeloid leukemia, Q692*, * represents a nonsense substitution at amino acid 692

Due to space limitation, we focus on the discussion of a somatic mutation on Tyr641 (Y641) of EZH2. This mutation was first reported in the whole genome sequencing of follicular and diffuse large B cell lymphomas [94]. Because Y641 is located in the SET domain, the in vitro enzymatic assay demonstrated that this mutant protein exhibited reduced methyltransferase activity. Surprisingly, the cancer cells harboring heterozygous Y641 mutation show hypertrimethylation of H3K27. This mystery was resolved by two elegant studies that demonstrated the unique interaction between wild-type and Y641-mutant EZH2 in catalytic reaction [95, 96]. Wild-type EZH2 displays high catalytic activity to induce me1 to H3K27. However, this enzyme shows low efficiency on the addition of me2 and me3. Interestingly, mutation of Y641 switches EZH2 to exhibit enhanced activity to catalyze me2 and me3 of H3K27. Cooperation between the wild-type and Y641-mutant EZH2 eventually increases trimethylation of H3K27 and the Y641 mutation is considered as a gain-of function mutation in follicular and diffuse large B-cell lymphomas. Although the biological consequence of Y641 mutation has been elucidated clearly, the contribution of many mutant modifiers to tumorigenesis is still unknown. For the mutations that occur at the enzymatic sites, the oncogenic function of the mutants can be attributed to the alteration of catalytic activity. However, many mutations are identified at the non-enzymatic sites. Whether these mutations are driver mutations needs further characterization.

### Targeting H3K27 modifiers in cancer therapy

Because EZH2 deregulation, notably overexpression, is frequently found in a variety of cancers, EZH2 has become an interesting therapeutic target for the development of anti-cancer drugs. The first EZH2 inhibitor described was 3-deazaneplanocin A (DZNep), a chemical inhibitor of *S*-adenosyl-*L*-homocysteine hydrolase, which induces the accumulation of the *S*-adenosyl-*L*-homocysteine to suppress the EZH2-mediated methylation via a feedback inhibition [120]. Subsequent high-throughput screening identified novel selective EZH2 inhibitors with potent anti-cancer effect in vitro and in experimental animals [121, 122]. In 2013, two orally active EZH2 inhibitors UNC1999 and EPZ-6438 were reported [123, 124]. Several elegant articles have already reviewed the development of the EZH2 inhibitors in detail [125–128]. As shown in Table 2, seven clinical trials registered in the ClinicalTrial.gov are actively enrolling patients or will begin to recruit patients this year. Among the four compounds currently undergoing clinical evaluation, EPZ-6438 (tazemetostat or E7438) progresses quickly and is now in three phase 2 clinical trials (NCT01897571, NCT02860286 and NCT02601950). Another phase 2 trial is an open-label extension (rollover) study to provide continuing availability of tazemetostat as a single agent to subjects who have completed their participation in antecedent studies so as to investigate the long-term safety profile and survival outcomes with tazemetostat (NCT02875548). Two novel EZH2 inhibitors CPI-1205 and GSK2816126 will undergo phase 1 trials. The structure and the inhibitory mechanism on EZH2 of CPI-1205 have not been disclosed yet. GSK2816126 (also known as GSK126) is a SAM structure analog and represses the EZH2 activity via substrate competition. In addition to direct inhibition of enzymatic activity, disruption of the PRC2 complex may also reduce the methylation of H3K27. This idea was previously confirmed by Kim et al. [129] who demonstrated that competitive peptides could block the interaction between EED and EZH2 and suppress EZH2-dependent tumor growth. Recently, the first EED peptide inhibitor MAP683 developed by Novartis will be tested for its safety and efficacy in diffuse large B-cell lymphoma.

**Table 2:** Cancer clinical trials of the inhibitors of the H3K27 methylation modifiers

Compound	Mechanism	Tumor type	Status	ClinicalTrials.gov identifier
CPI-1205	Unknown	B-cell lymphoma	Phase 1	NCT02395601
E7438 (EPZ-6438) (Tazemetostat)	SAM-competitive^a^	B-cell lymphoma Solid tumors Diffuse large cell lymphoma Follicular lymphoma Transformed follicular lymphoma Primary mediastinal large B-cell lymphoma	Phase 1 Phase 1 Phase 2 Phase 2	NCT01897571
Tazemetostat	SAM-competitive	Mesothelioma (with or without BAP1 deficiency)	Phase 2	NCT02860286
Tazemetostat	SAM-competitive	Rhabdoid tumors INI1-negative tumors Synovial sarcoma Malignant rhabdoid tumor of ovary	Phase 1	NCT02601937
Tazemetostat	SAM-competitive	Malignant rhabdoid tumors Rhabdoid tumors of the kidney Atypical teratoid rhabdoid tumors Selected tumors with rhabdoid features INI1-negative tumors Synovial sarcoma Malignant rhabdoid tumor of ovary Renal medullary carcinoma Epithelioid sarcoma	Phase 1	NCT02601950
GSK2816126	SAM-competitive	Diffuse large B cell lympho Transformed follicular lymphoma Non-Hodgkin’s lymphomas Solid tumors Multiple myeloma	Phase 1	NCT02082977
MAK683	EED inhibitor	Diffuse large B cell lymphoma	Phase 1/2	NCT02900651
Tazemetostat	SAM-competitive	Diffuse large B cell lymphoma Follicular lymphoma Malignant rhabdoid tumor Rhabdoid tumors of the kidney Atypical teratoid rhabdoid tumors Synovial sarcoma Epithelioid sarcoma Mesothelioma Advanced solid tumors	Phase 2	NCT02875548^b^

*Source*: http://www.clinicaltrials.gov

^a^ SAM: *S*-adenosyl-*L*-methionine

^b^ This study provides continuing availability to tazemetostat as a single agent to subjects who have completed their participation in an antecedent tazemetostat study

Whether H3K27 demethylases including KDM6A, KDM6B and KDM7A play an oncogenic or anti-oncogenic role in cancers is under active investigation. In addition, the effect of these demethylases on tumorigenesis may be cell context-dependent. While many studies demonstrate the mutations of KDM6A and KDM6B in cancers and suggest a tumor-suppressive function of these two demethylases [130–135], several studies show KDM6A and KDM6B may exhibit oncogenic activity [136, 137]. A recent study demonstrated that KDM6A is a co-activator of the oncogenic transcription factor TAL1 and is essential for disease progression of TAL1-positive T-cell acute lymphoblastic leukemia (T-ALL) [138]. However, a subsequent study showed that KDM6B is required for the initiation and maintenance of T-ALL, while KDM6A acts as a tumor suppressor in T-ALL and is frequently mutated in this cancer [130]. Two catechols screened from the natural product library were the first described inhibitors for KDM6A [139]. However, they also showed similar affinity to KDM4C. By using a structure-guided chemoproteomics approach, Kruidenier et al. [140] developed GSK-J4 as a selective pan-KDM6 inhibitor that targeted both KDM6A and KDM6B. In vivo administration of GSK-J4 killed the TAL1-positive primary human leukemia cells in a patient-derived xenotransplant (PDX) model [139]. Novel KDM6B inhibitors modified from the previously identified pan-KDM6 inhibitor GSK-J1 have been reported recently and the ethyl ester prodrugs of these inhibitors showed better activity than GSK-J4 in a cell-based functional assay [141].

However, it is noteworthy that GSK-J1/J4 may also inhibit KDM5B and KDM5C at higher concentrations [142]. Therefore, in addition to affecting H3K27 methylation by inhibiting KDM6A and KDM6B, these inhibitors may also influence H3K4 methylation. The biological function of KDM7A in tumorigenesis is largely unknown. Nutrition depletion upregulated KDM7A and suppressed tumor growth by inhibiting angiogenesis [143]. In addition, KDM7A is sensitive to 2-hydroxyglutarate (2-HG), an onco-metabolite produced by the mutant isocitrate dehydrogenase 1 (IDH) or IDH2, suggesting an anti-cancer role [144]. A hydroxamate analog has been demonstrated to inhibit KDM2A, KDM7A and KDM7B at micromolar range [145]. However, selective KDM7A inhibitors are still lacking.

In addition to directly targeting the H3K27-modifying enzymes, the identification of synthetically lethal genes or addictive genes in cancer cells with genetic alterations (mutations, overexpression or down-regulation) of H3K27 modifiers may provide another strategy for cancer therapy. For example, as aforementioned, EZH2 epigenetically suppresses autophagy-associated genes, which leads to hyperactivation of mTOR in colon cancer [86]. It is possible that EZH2-mutated or -overexpressed cancer cells may show higher sensitivity to the mTOR inhibitors.

## Conclusion

The regulation of H3K27 methylation has been intensively addressed in the past decades. Our understanding on this histone marker has great breakthroughs in (1) the study of H3K27 trimethylation by the PRC2 complex and (2) the development of novel epigenetic drugs targeting H3K27 methylation in recent years. Jiao and Liu demonstrated the crystal structures of the PRC2 complex isolated from Chaetomium thermophilum and proposed the allosterical change of an EZH2 motif to regulate the active center for catalyzing H3K27 trimethylation [146]. This study provides invaluable information for the interaction between histone marker and the PRC2 complex. The identification of the SAM competitor tazemetostat as an EZH2 inhibitor greatly promotes the development of novel agents targeting histone methyltransferases and demethylases. Tazemetostat is now undergoing various phase II clinical trials and the application of this EZH2 inhibitor in different cancers is expected in the coming years.
